# Gauging the learning environment at Damascus University Pharmacy School in Syria using the DREEM questionnaire: A cross-sectional study

**DOI:** 10.12688/mep.19333.1

**Published:** 2022-10-19

**Authors:** Ghaith Alfakhry, Rowaida Saymeh, Issam Jamous, Khaled Alhomsi

**Affiliations:** 1 Education Quality and Scientific Research Office, Al-Sham Private University, Damascus, Damascus Governorate, NA, Syria; 2 Program of Medical Education, Syrian Virtual University, Damascus, Damascus Governorate, NA, Syria; 3 Faculty of Dentistry, Damascus University, Damascus, Damascus Governorate, NA, Syria; 4 Department of Periodontology, Damascus University Faculty of Dentistry, Damascus, Damascus Governorate, NA, Syria; 5 Department of Fixed Prosthodontics, Damascus University Faculty of Dentistry, Damascus, Damascus Governorate, NA, Syria; 6 Scientific Research Office, Al-Sham Private University, Damascus, Damascus Governorate, NA, Syria

**Keywords:** Pharmacy education, undergraduate education, learning environment, quantitative study, DREEM questionnaire, developing country, Damascus University, Syria

## Abstract

**Introduction**: This study was undertaken to provide the first record of evaluation of the educational environment of the Bachelor of Pharmacy program at Damascus University (DU), Syria using the internationally adopted Dundee Ready Education Environment Measure (DREEM) tool and compare it with other pharmacy schools around the world.

**Methods:** A cross-sectional study was conducted at DU Pharmacy School in 2022. The validated DREEM 50-item inventory was added to Google Forms and used to collect data electronically. Data was collected during the second term between April 2022 and June 2022. Students from all years were included.

**Results:** A total of 269 students completed the questionnaire. The Cronbach’s alpha of the DREEM questionnaire was 0.94. The total DREEM score was 89.8±32.1/200. DU Pharmacy School scored significantly less on the total DREEM score than its other counterparts around the world with a large effect size ( *d*>0.80). All subscales scored below 50% and the lowest scoring subscales were students’ perception of learning (SPL=41.8%) and students’ perception of the social environment (SSP=42.5%).

**Conclusions:** This study provides the first assessment of the learning environment at DU Pharmacy School. The findings implied that the educational environment is in need of major improvement, especially in areas related to teaching and learning practices and the general social environment; future research should focus on designing interventions that could effectively address these potential areas of improvement..

## Introduction

In health profession education, the learning environment (LE) has an influence on students' academic achievement, professional development, and general well-being ( Enns *et al*., 2016; Tempski *et al*., 2015). The LE can be defined as students’ experience of their institutions' curriculum, facilities, and social interactions with colleagues and staff members ( Tackett *et al*., 2017). Since the LE revolves around students’ experience and perception, more and more attention is being given to students’ feedback as a key component in the assessment of the learning environment. Measuring the quality of the LE is the first step toward addressing issues that hinder students’ development. This evaluation builds a blueprint of the desirable LE that can enable students to excel academically, socially, and professionally.

Several methods and tools have been used to capture students’ perceptions of the LE. Some researchers adopted a qualitative approach while others adopted a quantitative questionnaire-based approach. The latter is advantageous especially when trying to quantify and compare the LE of different institutes ( Al-Hazimi *et al*., 2004). One of the most common questionnaire-based tools is the DREEM (Dundee Ready Education Environment Measure) ( Roff, 2005). This tool has been adopted and used globally across various healthcare fields including medicine, dentistry, nursing, and pharmacy ( Bashir *et al*., 2020; Miles *et al*., 2012). Other questionnaires have been used to measure the LE such as MEEM (Medical Education Environment Measure) and Health Education Learning Environment Survey (HELES) ( Pimparyon *et al*., 2000; Rusticus *et al*., 2020). However, DREEM is the most widely adopted measure of the LE in medical schools and other health professions ( Miles *et al*., 2012).

In Syria, pharmacy education is comprised of a 5-year term-based program that ends with students acquiring a Bachelor of Pharmacy (B.Pharm) degree ( Bahnassi, 2020). Enrolment in pharmacy schools is accessible only after completing secondary education and one preparatory year ( Bahnassi, 2020). In 2015, the preparatory year was made compulsory for all students wanting to enroll in one of the healthcare-related faculties which include medicine, dentistry, and pharmacy ( Bahnassi, 2020). After finishing the preparatory year, students become second-year pharmacy students. Damascus University (DU) Pharmacy School is considered one of the largest and oldest schools of its kind in Syria, hosting over 4000 students according to 2022 records. Pharmacy schools at DU and other Syrian universities still use traditional teaching practices ( Bahnassi, 2020). Additionally, Damascus University is the only university in the Arab World that maintained the use of the native Arabic language as the primary language of instruction in health profession education ( Alfakhry *et al*., 2020). In 2007, a curriculum reform at DU Pharmacy school was attempted with the help of international experts ( Kayyal & Gibbs, 2012); the reform aimed to modernize the curriculum to address society's needs and meet the expectations of stakeholders. However, by 2009, no progress was made by the assigned working groups and the curriculum reform was abandoned; some of the factors that hindered the curriculum reform were the low motivation for curriculum change within the University and poor knowledge in curriculum development and reform strategies among faculty members ( Kayyal & Gibbs, 2012). It is only normal to speculate that the outdated curriculum at DU Pharmacy School is not ideal for fostering a positive LE. The curriculum is not the only factor that could have affected the LE, the state of war Syria has been living in for the past decade has greatly also affected higher education and the Syrian universities' capacity to train a competent workforce ( Dashash, 2019). A study that evaluated stress factors at DU Dental School indicated some stressors that pertained to the educational environment ( Shehada *et al*., 2022). Nevertheless, the LE in DU pharmacy education is an area that was not investigated before. Therefore, this study aims to provide the first evaluation of the students’ perception of the learning environment at Damascus University Pharmacy School in 2022 using the internationally adopted DREEM questionnaire and compare it with other pharmacy schools around the world.

## Methods

### Study design

This is an observational cross-sectional study. By considering quantitative indicators pre-specified by the DREEM inventory, the learning environment at Damascus University Pharmacy School was evaluated.

### Participants and setting

Seven public universities in Syria offer Bachelor of Pharmacy (B.Pharm) degrees; the major university being Damascus University. Other universities have been greatly affected by the Syrian conflict, which led many pharmacy students to go to Damascus University which is in a relatively conflict-free zone. In this study, the defined population included all students at Damascus University Pharmacy School except for first-year students who could not be considered pharmacy students as they were still in the preparatory year. River sampling and snowball sampling (non-probability sampling methods) were used; social media platforms such as Facebook, WhatsApp and Telegram were used to approach participants who completed an anonymous electronic questionnaire designed on Google Forms. The research team tried to approach as many participants as possible and data collection stopped only after exhausting all efforts.

### Ethical considerations

Ethical approval for the study was granted by the Syrian Virtual University (SVU) no. 479/0 on 19 ^th ^April, 2022. Participation in the study was voluntary and anonymous. It was explained in the questionnaire introduction that the generated data will be used for research purposes only and to evaluate the quality of the learning environment at Damascus University Pharmacy School. Participants provided their consent to participate via answering yes-or-no consent in the electronic questionnaire.

### Data collection

The validated DREEM questionnaire was used; the questionnaire was anonymous and no identifying information was collected. This study used an Arabic version of the questionnaire which was reported and used in a previous study ( Al-Qahtani, 1999). A blank copy of the Arabic questionnaire that was used as well as an English version are available on Figshare ( Alfakhry *et al*., 2022a; Alfakhry *et al*., 2022b). Previous studies reported the validity and reliability of the DREEM questionnaire ( Roff, 2005; Roff *et al*., 1997). The DREEM questionnaire was used previously to evaluate the LE at pharmacy schools ( Wong *et al*., 2015). The DREEM questionnaire includes 50 closed-ended statements under 5 domains:

- Students’ perception of learning (SPL): 12 items, score out of 48
- Students’ perception of teachers (SPT): 11 items, score out of 44
- Students’ academic self-perceptions (ASP): 8 items, score out of 32
- Students’ perception of atmosphere (SPA): 12 items, score out of 48
- Students’ social self-perception (SSP): 7 items, score out of 28

Each item is scored on a 5-point rating scale: strongly disagree (0), disagree (1), unsure/doesn’t apply (2), agree (3), strongly agree (4). Reverse coding was done for negative items 4, 8, 9, 17, 25, 35, 39, 48, and 50 so that a higher value donates a more positive result. The interpretation of the DREEM scale and its subscales is illustrated in Table 1 according to McAleer & Roff (2002). As for the individual item, any item that scored below 2 was considered an area that needs attention. No identifying information was required to maintain anonymity. Google Forms was used to collect responses electronically via posting it on social media platform and inviting students to complete it. Google Forms settings were adjusted so that participants needed to fill in all items to submit their responses. Therefore, missing data was not encountered. No specific time-frame was set for participants to complete the questionnaire. Data collection started on 25 ^th ^April, 2022, and ended on 20 ^th ^June, 2022 when no more questionnaire forms were being filled.

**Table 1.** A guide for interpretating the DREEM scale and its subscales.

	Score	Interpretation
**Total DREEM**	0-50	Very poor
	51-100	Plenty of problems
	101-150	More positive than negative
	151-200	Excellent
**Subscales**		
**SPL**	0-12	Very poor
	13-24	Teaching is viewed negatively
	25-36	A more positive approach
	37-48	Teaching highly thought of
**SPT**	0-11	Abysmal
	12-22	In need of some retraining
	23-33	Moving in the right direction
	34-44	Model teachers
**ASP**	0-8	Feeling of total failure
	9-16	Many negative aspects
	17-24	Feeling more on the positive side
	25-32	Confident
**SPA**	0-12	A terrible environment
	13-24	There are many issues that need to be changed
	25-36	A more positive atmosphere
	37-48	A good feeling overall
**SSP**	0-7	Miserable
	8-14	Not a nice place
	15-21	Not very bad
	22-28	Very good socially

The DREEM questionnaire data of different universities around the world were also extracted from different studies ( Bashir *et al*., 2020; Brown *et al.*, 2011; Wong *et al.*, 2015). These universities included Taylor’s University in Malaysia, Cardiff University in the United Kingdom, Monash University in Australia, and an anonymous institute coded as 1 as reported in the cited study from Pakistan ( Bashir *et al*., 2020). University ranking data was extracted from the most recent record on QS World University Rankings ( QS World University Rankings).

### Data analysis

One-sample *t*-test was used to compare Damascus University Pharmacy School DREEM data with that of other pharmacy schools around the world. A *P*-value less than 0.05 was considered statistically significant. Cohen’s *d* was used as a measure of effect size; a *d* value between 0.2–0.5 is considered small, 0.5–0.8 is medium, and when it’s over 0.8, it’s considered large according to Cohen 1988 ( Cohen, 1988). Cronbach’s alpha was used as a measure of internal consistency of the used scale in the new environment. Google Forms was used to administer the questionnaire electronically; Microsoft Excel (2019) was used for data processing and IBM SPSS 26.0 (2020) was used to conduct statistical analysis.

## Results

A total number of 269 students completed the DREEM questionnaire; the sample age mean was 22.4 (SD=1.9); 82.2% (n=221) were female students. In terms of year of study, 147 were in their fifth year, 53 were fourth years, 41 were third-year students and 28 were second years. Cronbach’s alpha value for the DREEM 50-item was 0.94. The full dataset can be found under *Underlying data* ( Alfakhry *et al*., 2022a).

The overall DREEM score was 89.8/200. The DREEM scale and each of its subscales scores are illustrated in Table 2. All five subscales scored below 50%; SPL (41.8%); SPT (45.6%); ASP (48.7%); SPA (45.4%); SSP (42.5%). The percentage of negative items (which scored below 2.0) in each domain was as follows: 75% in SPL, 45% in SPT, 75% in ASP, 58% in SPA, and 57% in SSP.

**Table 2.** Mean scores for the DREEM scale and subscales at Damascus University Pharmacy School.

	Mean (SD)	Interpretation
SPL (max=48)	20.1 (8.4)	Teaching is viewed negatively
SPT (max=44)	20.1 (8.0)	In need of some retraining
ASP (max=32)	15.6 (6.4)	Many negative aspects
SPA (max=48)	21.8 (9.6)	There are many issues that need to be changed
SSP (max=28)	11.9 (4.9)	Not a nice place
DREEM total score (max=200)	89.8 (32.1)	Plenty of problems
	n=269	

Table 3 compares Damascus University Pharmacy School's learning environment with other universities as measured by the DREEM inventory. All universities scored higher than DU Pharmacy School in the total DREEM score with a significant margin ( *P*<0.001) and a large effect size.

**Table 3.** Comparison of DREEM scores between Damascus University Pharmacy School and that of other universities around the world ( Brown *et al.*, 2011; Bashir *et al.*, 2020; Wong *et al.*, 2015).

	Anonymous university		Taylor’s University		Cardiff University		Monash University	
Country	Pakistan		Malaysia		UK		Australia	
University World Ranking	---		284		166		57	
Degree	Pharm.D		B.Pharm		MPharm		B.Pharm	
	**Mean**	**Eff. Size**	**Mean**	**Eff. size**	**Mean**	**Eff. Size**	**Mean**	**Eff. Size**
SPL (max=48)	28.5	-1.11	32.34	-1.57	35.11	-2.15	30.9	-1.41
SPT (max=44)	23.43	-0.47	27.4	-0.98	32.6	-1.88	30.3	-1.43
ASP (max=32)	18.09	-0.41	20.5	-0.82	22.2	-1.22	21.7	-0.87
SPA (max=48)	26.77	-0.55	29.4	-0.85	35.4	-1.71	32.3	-1.13
SSP (max=28)	15.50	-0.83	18.0	-1.33	20.02	-1.93	18.1	-1.63
DREEM total score (max=200)	112.28	-0.78	127.7	-1.28	145.4	-2.09	135.4	-1.50
	n=163		n=62		n=194		n=116	

Eff. Size: effect size. Cohen’s d has been used as a measure of the effect size. Pharm D: Doctor of Pharmacy; B.Pharm: Bachelor of Pharmacy; MPharm: Master of Pharmacy. University World Ranking data are extracted from QS University World Ranking ( QS World University Rankings).

At the subscale level, all universities showed a significant difference in comparison to DU Pharmacy School ( *P*<0.001) at every subscale. Taylor’s University, Cardiff University, and Monash University showed a large effect size, whereas the Pakistani university showed a large effect size in SPL and SSP and medium effect size in SPT, ASP, and SPA.

At the individual item level, the majority of items (62%, 31/50) scored less than 2. Only two items scored more than 3: item n. 15 ( *I have good friends in this school*) and item n. 31 ( *I have learned a lot about empathy in my profession*). The mean scores of each item are shown in Table 4 in ascending order. The lowest mean scores were observed in item n. 3 ( *There is a good support system for students who get stressed*), item n. 4 ( *I am too tired to enjoy the courses*), item n. 16 ( *The teaching helps to develop my competence*), item n. 21 ( *I feel I am being well prepared for my profession*), and item n. 9 ( *The teachers are authoritarian*).

**Table 4.** Mean score for each DREEM item at Damascus University Pharmacy School categorized according to their domain with an ascending order.

Domain	Mean (SD)
**Students’ perception of learning (SPL)**	
16. The teaching helps to develop my competence	1.09 (1.2)
22. The teaching helps to develop my confidence	1.24 (1.3)
13. The teaching is student centered	1.36 (1.3)
44. The teaching encourages me to be an active learner	1.39 (1.3)
47. Long term learning is emphasized over short-term learning	1.46 (1.3)
48. The teaching is too teacher centered	1.65 (1.2)
38. I am clear about the learning objectives of the courses	1.71 (1.3)
25. The teaching over emphasizes factual learning	1.78 (1.3)
1. I am encouraged to participate during teaching sessions	1.79 (1.2)
24. The teaching time is utilized properly	2.01 (1.3)
7. The teaching is often stimulating	2.35 (1.4)
20. The teaching is well focused	2.36 (1.2)
**Students’ perception of teaching (SPT)**	
9. The teachers are authoritarian	1.14 (1.2)
39. The teachers get angry during teaching sessions	1.52 (1.3)
32. The teachers provide constructive criticism	1.62 (1.3)
8. The teachers ridicule the students	1.87 (1.4)
50. The students irritate the teachers	1.91 (1.3)
6. The teachers are patient with patients	2.00 (1.1)
29. The teachers are good at providing feedback to students	2.06 (1.3)
18. The teachers have good communication skills with patients	2.09 (1.0)
37. The teachers give clear examples	2.53 (1.2)
40. The teachers are well prepared for their teaching sessions	2.64 (1.2)
2. The teachers are knowledgeable	2.86 (1.0)
**Students’ academic self-perception (ASP)**	
21. I feel I am being well prepared for my profession	1.13 (1.2)
27. I am able to memorize all I need	1.65 (1.3)
45. Much of what I have to learn seems relevant to a career in healthcare	1.79 (1.4)
26. Last year’s work has been a good preparation for this year’s work	1.80 (1.3)
5. Learning strategies which worked for me before continue to work even now	1.87 (1.4)
41. My problem solving skills are being well developed	1.96 (1.3)
10. I am confident about my passing this year	2.36 (1.5)
31. I have learned a lot about empathy in my profession	3.11 (1.0)
**Student’s perception of the atmosphere (SPA)**	
42. The enjoyment outweighs the stress of the courses	1.23 (1.4)
43. The atmosphere motivates me as a learner	1.26 (1.3)
23. The atmosphere is relaxed during lectures	1.42 (1.3)
49. I feel I am able to ask the questions I want	1.54 (1.3)
30. There are opportunities for me to develop interpersonal skills	1.61 (1.3)
34. The atmosphere is relaxed during seminars/ tutorials	1.67 (1.3)
11. The atmosphere is relaxed during the clinical teaching	1.83 (1.0)
12. This school is well timetabled	2.10 (1.4)
17. Cheating is a problem in the school	2.15 (1.4)
35. I find my experience disappointing	2.15 (1.5)
33. I feel comfortable in teaching sessions socially	2.27 (1.4)
36. I am able to concentrate well	2.58 (1.2)
**Students’ social self-perception (SSP)**	
3. There is a good support system for students who get stressed	0.52 (0.9)
4. I am too tired to enjoy the courses	0.90 (1.1)
14. I am rarely bored on the courses	1.14 (1.4)
46. My accommodation in the school is pleasant	1.37 (1.0)
28. I seldom feel lonely	2.30 (1.4)
19. My social life is good	2.58 (1.3)
15. I have good friends in this school	3.14 (1.1)

Underlined scores indicate a negative interpretation (<2.00)

## Discussion

The current study aimed to provide the first record of evaluation of the learning environment of the Bachelor of Pharmacy program at Damascus University. The findings showed that the learning environment at DU Pharmacy School is perceived negatively according to the DREEM scale. The DREEM total score indicated that there are plenty of problems that need to be addressed. All DREEM subscales (SPL, SPT, ASP, SPA, SSP) were on the negative spectrum indicating that all areas of the learning environment require major improvements. The finding also showed that pharmacy students at DU did not feel that they are being prepared well for practicing after graduation, nor did they believe that the current teaching helps develop their competence. Further, DU Pharmacy School scored the lowest in the DREEM scale and subscale in comparison to other pharmacy schools around the world including Pakistan which has a similar human development index to Syria ( UNDP, 2020).

The Cronbach’s alpha of the 50-item DREEM scale indicated excellent internal consistency and this is consistent with other studies ( Dimoliatis *et al*., 2010; Roff *et al*., 1997). The DREEM tool has been used to measure the LE in many pharmacy schools around the world. In Pakistan, the DREEM total score ranged between 109 and 131.7 out of 200 ( Bashir *et al*., 2020; Memon *et al*., 2018); Taylor’s University in Malaysia ( Wong *et al*., 2015) scored 127.7/200, and in more prestigious universities such as Cardiff University (the UK) ( Wong *et al*., 2015) and Monash University (Australia) ( Brown *et al*., 2011), the DREEM total scores were 145.5/200 and 133.0/200 respectively. In contrast, DU Pharmacy School scored 89.8/200 on the DREEM scale which is significantly lower than its counterparts across the world.

Similar to another study in Pakistan ( Ezeala & Moleki, 2018), items n. 3 ( *There is a good support system for students who get stressed)* and n. 9 *(The teachers are authoritarian)* also indicated a negative result (lower than 2); however, item n. 16 ( *The teaching helps to develop my competence*), item n. 21 ( *I feel I am being well prepared for my profession*) indicated a positive result in Pakistani universities in contrast to current study findings. A previous study ( El-Hammadi, 2012) in Syria suggested that the graduate pharmacy programs at Damascus University do not seem to satisfy students’ ambitions which is concurrent with this study's findings as the low scores on items no. 21 and 45 indicate. The previous study was conducted in 2010–2011 which marks the start of the Syrian conflict ( El-Hammadi, 2012). At that time, the Syrian economy and living standards were in a much better state in comparison to 2022. Nevertheless, the results of the 2010 study and this 2022 study were similar as shown above. This implies that the problem might be associated with the curriculum of DU Pharmacy School rather than the general context ( Bahnassi, 2020; Kayyal & Gibbs, 2012). The majority of graduates from DU Pharmacy School work as community pharmacists which requires skills in general healthcare, pharmacy, pharmaceutical care, clinical pharmacy as well as business management skills ( Bahnassi, 2020). On the other hand, the current pharmacy education in Syria focuses on the pharmaceutical industry and sciences, and the clinical component of the curriculum is still underdeveloped ( Bahnassi, 2020). This could explain why most students did not feel that the pharmacy education they received prepared them well for the profession ( El-Hammadi, 2012). The SPL subscale was perceived most negatively in comparison to other subscales; this suggests that current teaching and learning practices are not contributing to forming a positive LE. Another area in the LE at DU Pharmacy School that needs more attention is the general atmosphere and the social environment which were perceived as stressful and demotivating by students. The lack of a psychosocial support system is one of the areas that need to be considered seriously. This system is of utmost importance especially in fragile contexts such as the one in Syria.

The negative DREEM score at DU Pharmacy School can be understood in the light of the traditional curriculum which is characterized by teacher-centeredness and emphasis on information gathering learning approach; all modules of the program are mandatory; teaching is delivered using traditional methods such as lecturing and lab sessions. All these characteristics are substantiated by the current study results. The massive number of students who study pharmacy at DU is another detrimental element that is bound to affect the quality of education ( Bahnassi, 2020; Shehada *et al*., 2022). Moreover, the considerable rise in pharmacy graduates at Damascus University in conjunction with the limited job opportunities further emphasizes the importance of extending the Syrian pharmacists’ role and not limiting it to community pharmacy businesses ( Bahnassi, 2020). However, this requires a new legislative framework that redefines the role of pharmacists in Syria along with a curriculum reform that puts forward clinical competence as a core component of the B.Pharm program ( Bahnassi, 2020). There are multiple implications for having a generally negative educational environment such as decreasing academic performance, lower student satisfaction, and higher stress levels and burnout ( Alhaffar *et al*., 2019; Dyrbye *et al*., 2020).

### Limitations and strengths

The predominant percentage of female respondents is one of the limitations of this study. However, it is known that most pharmacy students at DU are females ( El-Hammadi, 2012) and this still holds true at DU Pharmacy School. The non-probability sampling method and the small sample size in comparison to the population size are other areas of weakness that could affect the generalisability of the study findings. However, due to pragmatic reasons, collection of larger sample size and the use of random sampling were not feasible options. The data collection process faced a lot of obstacles due to the obstructive regulations and the lack of motivation of the school administration and faculty.

The significance of this study lies in that it is the first evaluation of the learning environment at DU Pharmacy School. This study could help raise awareness of the need for a total educational reform that has been advocated for decades now ( Kayyal & Gibbs, 2012). If a curriculum reform is attempted at DU Pharmacy School, this study could act as a baseline reading of the reality of the LE. Furthermore, it could help identify potential areas of improvement that need to be prioritized.

## Conclusions

The findings of this study suggest that the educational environment at DU Pharmacy School is in need of major improvements, especially in the areas related to teaching and learning, the social environment, and the general atmosphere as defined by the DREEM subscales. Future research should focus on designing effective interventions that could address some of the areas of improvement in the educational environment at DU Pharmacy School.

## Data Availability

Figshare: Associated dataset and DREEM questionnaire forms for the study conducted at Damascus University Pharmacy School in 2022. https://doi.org/10.6084/m9.figshare.21152476.v6 ( Alfakhry *et al*., 2022a) This project contains the following underlying data: Data File.sav (dataset generated from this study) This project contains the following extended data: English version of DREEM questionnaire Arabic version of DREEM questionnaire Data are available under the terms of the Creative Commons Attribution 4.0 International license (CC-BY 4.0).
