# Supplementary material for: Gauging the learning environment at Damascus University Pharmacy School in Syria using the DREEM questionnaire: A cross-sectional study
Source: MedEdPublish (2016). 2023 Aug 10;12:60. Originally published 2022 Oct 19. [Version 2] doi: 10.12688/mep.19333.2 (PMC10776964; doi:10.12688/mep.19333.2)
Supplement: Peer review history

# Reviewer report: Reviewer response for version 2

**DOI:** 10.21956/mep.21157.r35784

**Published:** 2024-01-09

**Authors:** P Ravi Shankar

**Affiliations:** 1 IMU Center for Education, International Medical University, Kuala Lumpur, Malaysia

Thanks for the invitation to review this important and interesting study. I would like to have more details on the ethical review process. The authors mention that the study was approved by the Syrian Virtual University. Is Damascus University Pharmacy School affiliated to this university? Also can the authors provide more details of the sampling methods. In my opinion as the sample size is very small caution should be exercised while making recommendations and suggestions based on this study. The study is well-written and presented.

Have any limitations of the research been acknowledged?

Yes

Is the work clearly and accurately presented and does it cite the current literature?

Yes

If applicable, is the statistical analysis and its interpretation appropriate?

I cannot comment. A qualified statistician is required.

Are all the source data underlying the results available to ensure full reproducibility?

Yes

Is the study design appropriate and is the work technically sound?

Yes

Are the conclusions drawn adequately supported by the results?

Yes

Are sufficient details of methods and analysis provided to allow replication by others?

Yes

Reviewer Expertise:

Health Professions Education, Small group learning, pharmacology and pharmacy education

I confirm that I have read this submission and believe that I have an appropriate level of expertise to confirm that it is of an acceptable scientific standard.

# Reviewer report: Reviewer response for version 2

**DOI:** 10.21956/mep.21157.r34489

**Published:** 2023-08-24

**Authors:** Marzia Lommi

**Affiliations:** 1 Local Health Authority Roma, Rome, Italy

I consider the new version approved.

Have any limitations of the research been acknowledged?

Yes

Is the work clearly and accurately presented and does it cite the current literature?

Partly

If applicable, is the statistical analysis and its interpretation appropriate?

Partly

Are all the source data underlying the results available to ensure full reproducibility?

Yes

Is the study design appropriate and is the work technically sound?

Yes

Are the conclusions drawn adequately supported by the results?

Partly

Are sufficient details of methods and analysis provided to allow replication by others?

Partly

Reviewer Expertise:

NA

I confirm that I have read this submission and believe that I have an appropriate level of expertise to confirm that it is of an acceptable scientific standard.

# Reviewer report: Reviewer response for version 1

**DOI:** 10.21956/mep.20717.r33611

**Published:** 2023-07-14

**Authors:** Marzia Lommi

**Affiliations:** 1 Local Health Authority Roma, Rome, Italy

It is a pleasure to read this research article.

 I would like to make some suggestions to the authors to make it clearer. 

 Abstract

 Add information to introduction and results, based on my suggestions.

 Keywords 

 Please, use if available MESH terms.

 Introduction

 DREEM is certainly an adopted tool for assessing the educational environment around the world and in Syria. I suggest the authors cite studies that have evaluated its content validity and psychometric properties in systematic reviews (with COSMIN or JOANNA BRIGGS methodology) to justify its use even more. This is to answer the question: why did I choose this particular instrument over others?

 Instruments

 After the section "participants and setting," describe the instrument used, its dimensions, psychometric properties, etc. I kindly ask the authors to divide the information regarding the instrument from that regarding the data collection procedure.

 Data collection

 Here I expect to find the procedures used for data collection. It would be helpful to give reasons why the data were collected at a particular time of the year. 

 Results

 For the sample under study I would include a table that would consider the year of the course as the selection variable. It would be interesting to see by course year how the DREEM scores also vary. I would also put their significance (Chi square, Anova depending on the variable being analyzed) in the table next to the variables under study. 

 Discussion

 Based on the suggested modification I would also discuss these results in light of the literature.

 Conclusion 

 I would add something more in light of the discussion and conclusions already provided For clinical practice, training, and research why are the results of this study important?

 Thank you to the authors and the journal for the opportunity to enrich knowledge on this topic.

Have any limitations of the research been acknowledged?

Yes

Is the work clearly and accurately presented and does it cite the current literature?

Partly

If applicable, is the statistical analysis and its interpretation appropriate?

Partly

Are all the source data underlying the results available to ensure full reproducibility?

Yes

Is the study design appropriate and is the work technically sound?

Yes

Are the conclusions drawn adequately supported by the results?

Partly

Are sufficient details of methods and analysis provided to allow replication by others?

Partly

Reviewer Expertise:

Public health, Gerontology, Educational learning, Training of health care personnel, Wound care, Self care, Quality of life

I confirm that I have read this submission and believe that I have an appropriate level of expertise to confirm that it is of an acceptable scientific standard, however I have significant reservations, as outlined above.

# Response

**Published:** 2023-07-26

**Authors:** Ghaith Alfakhry

**Affiliations:** Al-Sham Private University, Syria

*Marzia Lommi* *It is a pleasure to read this research article.* *I would like to make some suggestions to the authors to make it clearer.*

**Response: The authors would like to thank the reviewer for the insightful comments and constructive feedback that helped us improve the quality of the reporting of our findings.**  

*Abstract* *Add information to introduction and results, based on my suggestions.*

**Response: We would like to thank the reviewer for the feedback. The Abstract has been modified accordingly.** **Please see pg. 3**  

*Keywords* *Please, use if available MESH terms.*

**Response: We thank the reviewer for this accurate feedback. We have revised the first three keywords and replaced them with their equivalent MeSH terms (** **education; pharmacy, undergraduate). However, the rest of the terms are not available on the MeSH, therefore, we had to use them as they are to make it easier to find our research for the larger community.** **Please see pg. 2, line 21:** **“Keywords:** **education; pharmacy, undergraduate; learning environment; Dundee Ready Educational Environment Measure; DREEM; Damascus University; Syria.”**  

*Introduction* *DREEM is certainly an adopted tool for assessing the educational environment around the world and in Syria. I suggest the authors cite studies that have evaluated its content validity and psychometric properties in systematic reviews (with COSMIN or JOANNA BRIGGS methodology) to justify its use even more. This is to answer the question: why did I choose this particular instrument over others?*

**Response: We would like to thank the reviewer for bringing to our attention this important point. After a through literature review, we have added the following to address the reviewer recommendation (content validity, psychometric properties of DREEM and the justification for the use of DREEM).** **Please see pg. 4, line 22-38:** **“A review revealed that DREEM showed to be consistently reliable across countries in comparison to 11 other educational environment measures and hence recommended the use of DREEM as the best measure for the educational environment in undergraduate settings** **(Soemantri et al., 2010)** **. Another review study revealed that although the DREEM inventory showed some content validity evidence, it compared unfavourably to other tools** **(Colbert-Getz et al., 2014)** **; nonetheless, its face and content validity in measuring the learning environment in pharmacy undergraduate settings has been supported by one study** **(Wong et al., 2015)** **. Psychometric property evaluation of the DREEM instrument has been conducted in multiple studies in various settings, some supported the construct validity of the instrument** **(Rotthoff et al., 2011)** **while others did not** **(Yusoff, 2012)** **. One study in particular showed that the internal consistency of the five subscales can be quite variable** **(Hammond et al., 2012)** **. Notwithstanding these shortcomings, the wide use of DREEM instrument globally does encourage its use so that comparisons could be made between different studies** **(Colbert-Getz et al., 2014)** **. Additionally, DREEM score has shown positive correlates with academic achievement, quality of life, positive attitude towards the program, less psychological distress and greater social support** **(Chan et al., 2018)** **. All of these factors encourage the use of the DREEM inventory.** **”**  

*Instruments* *After the section "participants and setting," describe the instrument used, its dimensions,* *psychometric properties, etc. I kindly ask the authors to divide the information regarding the* *instrument from that regarding the data collection procedure.*

**Response: We would like to thank the reviewer for helping organize our manuscript better. We have described the instrument used, its dimensions and psychometric properties in detail:** **Please see** **Data collection instrument: DREEM questionnaire, pg. 6, line 94-112** *:* **“ *Data collection instrument : DREEM questionnaire*** **The validated DREEM questionnaire was used; the questionnaire was anonymous and no identifying information was collected. This study used an Arabic version of the questionnaire which was reported and used in a previous study** **(Al-Qahtani, 1999)** **. A blank copy of the Arabic questionnaire that was used as well as an English version were provided on figshare** **(Alfakhry et al., 2022a, Alfakhry et al., 2022b)** **. Previous studies reported the validity and reliability of the DREEM questionnaire** **(Roff et al., 1997, Roff, 2005)** **. The internal consistency of DREEM as measured by the Cronbach’s alpha measure was over 0.90 in study conducted in 2023 on a sample of Syrian medical and dental students** **(Alfakhry et al., 2023a, Alfakhry et al., 2023b)** **. Additionally, the DREEM questionnaire was used previously to evaluate the LE at pharmacy schools** **(Wong et al., 2015, Bashir et al., 2020)** **. The DREEM questionnaire includes 50 closed-ended statements under 5 dimensions which are supported by factor analysis of one study** **(Rotthoff et al., 2011)** **and the qualitative validation during its development** **(Roff et al., 1997)** **: **

- **Students’ perception of learning (SPL): 12 items, score out of 48**
- **Students’ perception of teachers (SPT): 11 items, score out of 44**
- **Students’ academic self-perceptions (ASP): 8 items, score out of 32**
- **Students’ perception of atmosphere (SPA): 12 items, score out of 48**
- **Students’ social self-perception (SSP): 7 items, score out of 28”**

 
**Response: We have also revised this section as advised and put other sub-headings to separate the instrument description section and the data collection procedure section.** **Please see the sub-headings in the following page. 6-8:** ***“Data collection instrument: DREEM questionnaire*** ***Sampling method and data collection procedure*** ***Sampling method and data collection procedure*** ***DREEM data of other international universities”***  

*Data collection* *Here I expect to find the procedures used for data collection. It would be helpful to give reasons* *why the data were collected at a particular time of the year.*

**Response: The authors would like to extend their thanks to the reviewer for the helpful comments. We have now added a subheading entitled “** ***Sampling method and data collection procedure”*** **(pg. 6, line 119-124) and explained the data collection procedure in detail as requested. As for why the data collection occurred at the particular time mentioned in the paper (between April and June), it is because we considered that the beginning of the term was late March and second semester final exams occur in July. Therefore, it’s reasonable to collect data after students settle in and before they get occupied with preparing for their exams.** **This is now highlighted in pg. 6, line 131-134:** **“Data collection occurred at this particular time because it is a bit after the beginning of the second term which starts late March to allow students time to settle in with the new term and before students become occupied with the final exams which take place in July.”**  

*Results* *For the sample under study I would include a table that would consider the year of the course as* *the selection variable. It would be interesting to see by course year how the DREEM scores also* *I would also put their significance (Chi square, Anova depending on the variable being* *analyzed) in the table next to the variables under study.*

**Response: We would like to thank the reviewer for recommending comparing students’ perceptions of different years of study. We have now added a table a paragraph to address this point.** **Please see pg. 9, line 171-174:** **“Table 3 shows the scores of participants according to their year of study; one-way ANOVA showed a significant between different groups and post-hoc demonstrated that it was Year 2 students who had significantly higher scores than their senior counterparts.”**

**Table 3.** Mean score with the standard deviation according to the year of study. **Year of study** **One-way ANOVA**   Year 2 (n=28) Year 3 (n=41) Year 4 (n=53) Year 5 (n=147) *P*-value **SPL (max=48)** 25.3±7.7 19.4±7.5 19.0±9.1 19.8±8.2 0.007 **SPT (max=44)** 24.2±8.3 20.1±8.3 20.1±8.0 19.4±7.7 0.036 **ASP (max=32)** 17.8±5.1 13.3±6.1 14.5±6.7 16.3±6.3 0.008 **SPA (max=48)** 27.7±7.2 20.9±9.7 21.3±9.8 21.0±9.5 0.007 **SSP (max=28)** 14.8±4.2 11.4±4.9 11.6±5.5 11.6±4.6 0.011 **DREEM (max=200)** 110.0±27.7 58.3±30.7 86.7±34.3 88.2±31.4 0.005 Underlined cells indicate a value of negative interpretation. *Discussion* *Based on the suggested modification I would also discuss these results in light of the literature.*

**Response: We would like to thank the reviewer for the suggestions. We have now discussed the differences in scores between students of different years of study in the discussion.** **Please see pg. 14, line 218-221:** **“More senior participants seemed to display more negative perception of the learning environment in comparison to their younger counterparts. This finding seems to be consistent across disciplines, whether medical, dental, nursing according to one systematic review** **(Chan et al., 2018)** **and even pharmacy education** **(Bashir et al., 2020)** **.** **"**  

*Conclusion* *I would add something more in light of the discussion and conclusions already provided For* *clinical practice, training, and research why are the results of this study important?*

**Response: We would like to thank the reviewer for the insightful feedback. The conclusion has been rewritten in accordance with the comments provided.** **Please see pg. 15-16, line: 287-297:** **“Conclusion** **The findings of this study suggest that the educational environment at DU Pharmacy School is in need of major improvements, especially in the areas related to teaching and learning, the social environment, and the general atmosphere as defined by the DREEM subscales. Failure to improve the learning environment might hinder skills attainment necessary for practice post graduation. The faculty should consider reforming its curriculum and add more modules which help students meet their job demands after graduation in Syria whose job market especially focuses on clinical community pharmacy. Delivery of teaching should focus more students and shift the paradigm of teacher-centered education to student-centered. This study provides a quality improvement blueprint that future interventional research could use preciously address the areas that need most attention at DU Pharmacy School.”**  

*Thank you to the authors and the journal for the opportunity to enrich knowledge on this topic.*

**Response: It is us the authors who would like to express their sincere thanks to the reviewer’s time, effort, and honest dedication to help us improve the quality of our manuscript.**  

**References:**  

 AL-QAHTANI, M. F. M. 1999. *Approaches to study and learning environment in medical schools with special reference to the Gulf countries.* University of Dundee Dundee, UK.

 ALFAKHRY, G., MUSTAFA, K., ALMUKHALLALATI, A., ALHOMSI, K., SAYMEH, R. & JAMOUS, I. 2023a. Evaluation of the Undergraduate Learning Environment at Dental Schools in Syria. *International Dental Journal*.

 ALFAKHRY, G., NAEEM, A., ABOHAJAR, M. B., ALFAKHRY, A., MOHANDES, A. F., ALI, I., MAKHOUL, E., AHMED, N., ABLA, M. M. & ALHOMSI, K. 2023b. Revealing the significant shortcomings in the learning environment at the three largest medical schools in Syria: what’s next? *BMC Medical Education,* 23 **,** 1-10.

 ALFAKHRY, G., ROWAIDA, S., JAMOUS, I. & ALHOMSI, K. 2022a. Associated dataset and DREEM questionnaire forms for the study conducted at Damascus University Pharmacy School in 2022. figshare. Dataset. https://doi.org/10.6084/m9.figshare.21152476.v6

 ALFAKHRY, G., ROWAIDA, S., JAMOUS, I. & ALHOMSI, K. 2022b. English and Arabic versions of the used Dundee Ready Education Environment Measure (DREEM) questionnaire for the study conducted at Damascus University Pharmacy School and submitted to MedEdPublish. figshare. Dataset. https://doi.org/10.6084/m9.figshare.21257319.v1

 AMINU, I. M. & SHARIFF, M. N. M. 2014. Strategic orientation, access to finance, business environment and SMEs performance in Nigeria: Data screening and preliminary analysis. *European Journal of Business and Management,* 6 **,** 124-132.

 BASHIR, S., KHAN, A.-U., MAHMOOD, M., ABBAS, M., AKHTAR, S., KHAN, A. W., AQEEL, M. T., AKHLAQ, M. & AHMAD, H. 2020. Students’ perception of the learning environment in private-sector pharmacy institutes of Pakistan. *Pharmacy Education,* 20 **,** 191-197.

 CHAN, C. Y. W., SUM, M. Y., TAN, G. M. Y., TOR, P.-C. & SIM, K. 2018. Adoption and correlates of the Dundee Ready Educational Environment Measure (DREEM) in the evaluation of undergraduate learning environments–a systematic review. *Medical teacher,* 40 **,** 1240-1247.

 COHEN, J. 1988. Statistical power analysis for the behavioural sciences. Hillsdale, New Jersey: L. *Lea,* 56 **,** 102.

 COLBERT-GETZ, J. M., KIM, S., GOODE, V. H., SHOCHET, R. B. & WRIGHT, S. M. 2014. Assessing medical students’ and residents’ perceptions of the learning environment: exploring validity evidence for the interpretation of scores from existing tools. *Academic Medicine,* 89 **,** 1687-1693.

 GEORGE, D. & MALLERY, M. 2010. SPSS for windows step bysstep: A simple guide and reference.

 HAMMOND, S. M., O'ROURKE, M., KELLY, M., BENNETT, D. & O'FLYNN, S. 2012. A psychometric appraisal of the DREEM. *BMC medical education,* 12 **,** 1-5.

 ROFF, S. 2005. The Dundee Ready Educational Environment Measure (DREEM)—a generic instrument for measuring students’ perceptions of undergraduate health professions curricula. *Medical teacher,* 27 **,** 322-325.

 ROFF, S., MCALEER, S., HARDEN, R. M., AL-QAHTANI, M., AHMED, A. U., DEZA, H., GROENEN, G. & PRIMPARYON, P. 1997. Development and validation of the Dundee ready education environment measure (DREEM). *Medical teacher,* 19 **,** 295-299.

 ROTTHOFF, T., OSTAPCZUK, M. S., DE BRUIN, J., DECKING, U., SCHNEIDER, M. & RITZ-TIMME, S. 2011. Assessing the learning environment of a faculty: psychometric validation of the German version of the Dundee Ready Education Environment Measure with students and teachers. *Medical teacher,* 33 **,** e624-e636.

 SOEMANTRI, D., HERRERA, C. & RIQUELME, A. 2010. Measuring the educational environment in health professions studies: a systematic review. *Medical teacher,* 32 **,** 947-952.

 SULLIVAN, G. M. & FEINN, R. 2012. Using effect size—or why the P value is not enough. *Journal of graduate medical education,* 4 **,** 279-282.

 WONG, P. N., JOHN, D. N., DESLANDES, R. E. & HUGHES, M. L. 2015. Same syllabus, different country–using DREEM to compare the educational environments at two Pharmacy schools. *Pharmacy Education,* 15.

 YUSOFF, M. S. B. 2012. Psychometric properties of DREEM in a sample of Malaysian medical students. *Medical Teacher,* 34 **,** 595-596.

# Reviewer report: Reviewer response for version 1

**DOI:** 10.21956/mep.20717.r32742

**Published:** 2022-11-23

**Authors:** Andrea Manfrin

**Affiliations:** 1 School of Pharmacy and Biomedical Sciences, University of Central Lancashire, Preston, UK

Dear Editor and Authors, thank you for allowing me to review this exciting study.

 It is a significant study presenting the results of research conducted at Damascus University. The research provides the first evaluation of the Bachelor of Pharmacy programme adopting the Dundee Ready Education Environment Measure (DREEM) and comparing their results with other pharmacy schools worldwide.

 The findings suggest the need for significant improvement, focusing on the area related to teaching and learning practice and the social environment.

 This work is accurately presented and fits well with the current literature.

 Study design:

 The study has an appropriate design. However, I suggest adding the response rate to the abstract and result section. The paper indicated that there were over 4,000 students “according to 2022 records. **Therefore, the response rate should be 6.7% (**269/4000)

 As suggested by the authors, it is one of the limitations of the generalizability of the results.

 Data analysis:

 The method is clearly described providing all the relevant details. Nonetheless, this study is not hypothesis testing; therefore, the sample calculation was not required.

 It seems the authors considered the data normally distributed and performed the student t-test analysis providing means and SDs.

 Looking at the results and the scores, which are not providing a positive outlook, the overall score was 89.8/200 (plenty of problems), and the score for each domain was well below the average; the data suggests a skewed distribution which could be non-normal.

 The authors did not mention in the statistical analysis plan that they assessed the data's normality. I wonder whether a normality test could confirm a skewed distribution and, therefore, the requirement to use a non-parametric analysis.

 Please provide this crucial information or sustain your approach using an evidence-based argument.

 Table 3:

 This table compares Damascus University School of Pharmacy and the other four schools. Nevertheless, the Damascus University School of Pharmacy is not embedded in the table but lists only the other schools.

 I suggest redrafting this table and adding the Damascus University School of Pharmacy as a comparator. Then the table will become clearer and easy to understand.

 Table 3 shows that all Cohen d values are negative, but their interpretation is not included in the data analysis plan. Please revise.

 Limitations:

 The percentage of responses could also help contextualise this study's generalizability.

 Conclusions:

 The conclusions support the results, but the non-normality of the data could change the data analysis, the results (partially), and the discussion section slightly. Still, it should not change the overall message of this study which is very clearly presented.

Have any limitations of the research been acknowledged?

Yes

Is the work clearly and accurately presented and does it cite the current literature?

Yes

If applicable, is the statistical analysis and its interpretation appropriate?

Partly

Are all the source data underlying the results available to ensure full reproducibility?

Yes

Is the study design appropriate and is the work technically sound?

Yes

Are the conclusions drawn adequately supported by the results?

Yes

Are sufficient details of methods and analysis provided to allow replication by others?

Partly

Reviewer Expertise:

Health Services Research, Pedagogic Research, Advanced Statistics

I confirm that I have read this submission and believe that I have an appropriate level of expertise to confirm that it is of an acceptable scientific standard, however I have significant reservations, as outlined above.

# Response

**Published:** 2023-07-26

**Authors:** Ghaith Alfakhry

**Affiliations:** Al-Sham Private University, Syria

*Andrea Manfrin* *School of Pharmacy and Biomedical Sciences, University of Central Lancashire, Preston, UK* *Dear Editor and Authors, thank you for allowing me to review this exciting study.* *It is a significant study presenting the results of research conducted at Damascus University. The research provides the first evaluation of the Bachelor of Pharmacy programme adopting the Dundee Ready Education Environment Measure (DREEM) and comparing their results with other pharmacy schools worldwide. The findings suggest the need for significant improvement, focusing on the area related to teaching and learning practice and the social environment. This work is accurately presented and fits well with the current literature.*

**Response: The authors would like to express their sincere appreciation to the reviewer for the constructive and complimenting feedback on our work.**  

*Study design:* *The study has an appropriate design. However, I suggest adding the response rate to the abstract and result section. The paper indicated that there were over 4,000 students “according to 2022 records. Therefore, the response rate should be 6.7% (269/4000). As suggested by the authors, it is one of the limitations of the generalizability of the results.*

**Response: We would like to thank the reviewer for pointing this out. Although we agree with the reviewer in indicating that our sample is about 6.7% of the total population, we would not call it “response rate.” Response rate insinuates that we have reached out to each and every pharmacy student at Damascus University; we are not sure that the electronic survey reached all students, and we would be at fault in assuming that. Therefore, we will simply point out that our sample which is consistent of 269 students form 6.7% of the total population. We have added this information to both the abstract and results sections.** **Please see Abstract pg. 3** **“Abstract: Results:  A total of 269 students completed the questionnaire; that is about 6.7% of the total population.”** **and Results, pg. 9, line 161-162** **“A total number of 269 students completed the DREEM questionnaire; that is about 6.7% (269/4000) of the total population.”**  

*Data analysis:* *The method is clearly described providing all the relevant details. Nonetheless, this study is not* *hypothesis testing; therefore, the sample calculation was not required.*

**Response: We would like to thank the reviewer for the comment. We only set the *P* value at 0.05 not for hypothesis testing but rather comparative purposes when using the t-test. The effect size on the other hand was used as a measure of the standardized difference between two means and to complement the information given by the p-value. It is important to mention the effect size along with the p-value, and this is supported by a previous paper in medical education research** **(Sullivan and Feinn, 2012)** **:** **“The effect size is the main finding of a quantitative study. While a P value can inform the reader whether an effect exists, the P value will not reveal the size of the effect. In reporting and interpreting studies, both the substantive significance (effect size) and statistical significance ( *P*-value) are essential results to be reported.”**  

*It seems the authors considered the data normally distributed and performed the student t-test analysis providing means and SDs.* *Looking at the results and the scores, which are not providing a positive outlook, the overall score was 89.8/200 (plenty of problems), and the score for each domain was well below the average; the data suggests a skewed distribution which could be non-normal.* *The authors did not mention in the statistical analysis plan that they assessed the data's normality. I wonder whether a normality test could confirm a skewed distribution and, therefore, the requirement to use a non-parametric analysis.* *Please provide this crucial information or sustain your approach using an evidence-based argument.*

**Response: We would like to thank the reviewer for giving us the chance to elaborate on this important aspect. We have actually considered the normality of data before conducting the t-test. We used Skewness and Kurtosis as measures of data normality: please see the following studies:** **Hair Jr, J. F., Black, J. W., Babin, B. J., & Anderson, E. R. (2010).** **Multivariate Data Analysis** **(Seventh Ed., pp.** **1–758). Edinburgh: Pearson Education Limited.** **Kline, R. B. (2011).** **Principles and Practice of Structural Equation Modeling** **(5th ed., pp. 3–427). New York:** **The Guilford Press** **Tabachnick, B. G., & Fidell, L. S. (2013).** **Using Multivariate Statistics** **(6th ed., pp. 1–983). New Jersey:** **Pearson Education Inc** **It has been suggested by Kline (2011) that if skewness is greater than 3 and Kurtosis is greater than 10, there might be a problem in data normality; however, our values  (see the table below) is well below that cut-off point, supporting the normal distribution assumption. Moreover, Tabachnick and Fidell (2013) state that if the sample size is larger than 200 (our sample size is 269), deviation from normality of skewness and kurtosis often do not make real difference in the analysis.** **Skewness** **Kurtosis** **SPL** **0.33** **-0.53** **SPT** **0.13** **-0.42** **ASP** **0.07** **-0.44** **SPA** **0.18** **-0.50** **SSP** **-0.14** **-0.20** **DREEM** **0.26** **-0.22**  

**We have now mentioned that data normality has been checked and confirmed in the manuscript in the Methods section, pg. 8, line: 146-147:** **“** **Data normality has been checked and confirmed using skewness and Kurtosis; therefore, parametric tests were conducted.”**  

*Table 3:* *This table compares Damascus University School of Pharmacy and the other four schools.* *Nevertheless, the Damascus University School of Pharmacy is not embedded in the table but lists only the other schools. I suggest redrafting this table and adding the Damascus University School of Pharmacy as a comparator. Then the table will become clearer and easy to understand.*

**Response: We would like to thank the reviewer for this insightful suggestion. We have now added Damascus University School of Pharmacy data to Table 4 as you have suggested.  (Table 3 has been changed to table 4 after an additional table has been added (table 3))** **Please see Table 4, pg. 10:** **Damascus University (comparator)** **Anonymous university** **Taylor’s University** **Cardiff University** **Monash University**

 Country Syria Pakistan Malaysia UK Australia University World Ranking 1201-1400 --- 284 =154 42 Degree B. Pharm Pharm.D B.Pharm MPharm B.Pharm    

**Mean** **Eff. Size** **Mean** **Eff. size** **Mean** **Eff. Size** **Mean** **Eff. Size** SPL (max=48) 20.1 (8.4) 28.5 -1.11 32.34 -1.57 35.11 -2.15 30.9 -1.41 SPT (max=44) 20.1 (8.0) 23.43 -0.47 27.4 -0.98 32.6 -1.88 30.3 -1.43 ASP (max=32) 15.6 (6.4) 18.09 -0.41 20.5 -0.82 22.2 -1.22 21.7 -0.87 SPA (max=48) 21.8 (9.6) 26.77 -0.55 29.4 -0.85 35.4 -1.71 32.3 -1.13 SSP (max=28) 11.9 (4.9) 15.50 -0.83 18.0 -1.33 20.02 -1.93 18.1 -1.63 DREEM total score (max=200) 89.8 (32.1) 112.28 -0.78 127.7 -1.28 145.4 -2.09 135.4 -1.50   N=269 n=163 n=62 n=194 n=116        

*Table 3 shows that all Cohen d values are negative, but their interpretation is not included in the* *data analysis plan. Please revise.*

**Response: We would like to thank the reviewer for making us aware of this point. We have now added an interpretation of the effect size values in the data analysis plan. Please see pg. 8, line: 150-154:** **“The effect size (Cohen’s d) can be either negative or positive depending on the direction of the comparison. If the effect size is negative, this indicates that the first mean is smaller than the second one. This can be deduced from Cohen’s d formula which is as follows: M1-M2/SD** **(Cohen, 1988)** **.The negative effect size value is interpreted just as the positive one.** **”**    

*Limitations:* *The percentage of responses could also help contextualize this study's generalizability.*

**Response: We would like to thank the reviewer for the suggestion. We have now added the percentage of our sample in comparison to the whole population size in the limitations section. Please see pg. 15, Line 276:** **“The non-probability sampling method and the small sample size in comparison to the population size (6.7%) are other areas of weakness that could affect the generalisability of the study findings.”**  

*Conclusions:* *The conclusions support the results, but the non-normality of the data could change the data* *analysis, the results (partially), and the discussion section slightly. Still, it should not change the* *overall message of this study which is very clearly presented.*

**Response: We would like to thank the reviewer for the constructive feedback. The reviewer might agree with us that since the normality assumption was met, the results and discussion section can remain unchanged. However, we have made slight changes to the conclusion according to second reviewer’s suggestions.** **Please see pg. 15-16, line 287-297:** **“Conclusions** **The findings of this study suggest that the educational environment at DU Pharmacy School is in need of major improvements, especially in the areas related to teaching and learning, the social environment, and the general atmosphere as defined by the DREEM subscales. Failure to improve the learning environment might hinder skills attainment necessary for practice post graduation. The faculty should consider reforming its curriculum and add more modules which help students meet their job demands after graduation in Syria whose job market especially focuses on clinical community pharmacy. Delivery of teaching should focus more students and shift the paradigm of teacher-centered education to student-centered. This study provides a quality improvement blueprint that future interventional research could use preciously address the areas that need most attention at DU Pharmacy School. “**    

**References**

 AL-QAHTANI, M. F. M. 1999. *Approaches to study and learning environment in medical schools with special reference to the Gulf countries.* University of Dundee Dundee, UK.

 ALFAKHRY, G., MUSTAFA, K., ALMUKHALLALATI, A., ALHOMSI, K., SAYMEH, R. & JAMOUS, I. 2023a. Evaluation of the Undergraduate Learning Environment at Dental Schools in Syria. *International Dental Journal*. ALFAKHRY, G., NAEEM, A., ABOHAJAR, M. B.,

 ALFAKHRY, A., MOHANDES, A. F., ALI, I., MAKHOUL, E., AHMED, N., ABLA, M. M. & ALHOMSI, K. 2023b. Revealing the significant shortcomings in the learning environment at the three largest medical schools in Syria: what’s next? *BMC Medical Education,* 23 **,** 1-10.

 ALFAKHRY, G., ROWAIDA, S., JAMOUS, I. & ALHOMSI, K. 2022a. Associated dataset and DREEM questionnaire forms for the study conducted at Damascus University Pharmacy School in 2022. figshare. Dataset. https://doi.org/10.6084/m9.figshare.21152476.v6

 ALFAKHRY, G., ROWAIDA, S., JAMOUS, I. & ALHOMSI, K. 2022b. English and Arabic versions of the used Dundee Ready Education Environment Measure (DREEM) questionnaire for the study conducted at Damascus University Pharmacy School and submitted to MedEdPublish. figshare. Dataset. https://doi.org/10.6084/m9.figshare.21257319.v1

 AMINU, I. M. & SHARIFF, M. N. M. 2014. Strategic orientation, access to finance, business environment and SMEs performance in Nigeria: Data screening and preliminary analysis. *European Journal of Business and Management,* 6 **,** 124-132.

 BASHIR, S., KHAN, A.-U., MAHMOOD, M., ABBAS, M., AKHTAR, S., KHAN, A. W., AQEEL, M. T., AKHLAQ, M. & AHMAD, H. 2020. Students’ perception of the learning environment in private-sector pharmacy institutes of Pakistan. *Pharmacy Education,* 20 **,** 191-197.

 CHAN, C. Y. W., SUM, M. Y., TAN, G. M. Y., TOR, P.-C. & SIM, K. 2018. Adoption and correlates of the Dundee Ready Educational Environment Measure (DREEM) in the evaluation of undergraduate learning environments–a systematic review. *Medical teacher,* 40 **,** 1240-1247.

 COHEN, J. 1988. Statistical power analysis for the behavioural sciences. Hillsdale, New Jersey: L. *Lea,* 56 **,** 102.

 COLBERT-GETZ, J. M., KIM, S., GOODE, V. H., SHOCHET, R. B. & WRIGHT, S. M. 2014. Assessing medical students’ and residents’ perceptions of the learning environment: exploring validity evidence for the interpretation of scores from existing tools. *Academic Medicine,* 89 **,** 1687-1693.

 GEORGE, D. & MALLERY, M. 2010. SPSS for windows step bysstep: A simple guide and reference.

 HAMMOND, S. M., O'ROURKE, M., KELLY, M., BENNETT, D. & O'FLYNN, S. 2012. A psychometric appraisal of the DREEM. *BMC medical education,* 12 **,** 1-5.

 ROFF, S. 2005. The Dundee Ready Educational Environment Measure (DREEM)—a generic instrument for measuring students’ perceptions of undergraduate health professions curricula. *Medical teacher,* 27 **,** 322-325.

 ROFF, S., MCALEER, S., HARDEN, R. M., AL-QAHTANI, M., AHMED, A. U., DEZA, H., GROENEN, G. & PRIMPARYON, P. 1997. Development and validation of the Dundee ready education environment measure (DREEM). *Medical teacher,* 19 **,** 295-299.

 ROTTHOFF, T., OSTAPCZUK, M. S., DE BRUIN, J., DECKING, U., SCHNEIDER, M. & RITZ-TIMME, S. 2011. Assessing the learning environment of a faculty: psychometric validation of the German version of the Dundee Ready Education Environment Measure with students and teachers. *Medical teacher,* 33 **,** e624-e636.

 SOEMANTRI, D., HERRERA, C. & RIQUELME, A. 2010. Measuring the educational environment in health professions studies: a systematic review. *Medical teacher,* 32 **,** 947-952.

 SULLIVAN, G. M. & FEINN, R. 2012. Using effect size—or why the P value is not enough. *Journal of graduate medical education,* 4 **,** 279-282.

 ONG, P. N., JOHN, D. N., DESLANDES, R. E. & HUGHES, M. L. 2015. Same syllabus, different country–using DREEM to compare the educational environments at two Pharmacy schools. *Pharmacy Education,* 15.

 YUSOFF, M. S. B. 2012. Psychometric properties of DREEM in a sample of Malaysian medical students. *Medical Teacher,* 34 **,** 595-596.
